# Preparation and Gas Sensing Properties of PANI/SnO_2_ Hybrid Material

**DOI:** 10.3390/polym13091360

**Published:** 2021-04-21

**Authors:** Qiaohua Feng, Huanhuan Zhang, Yunbo Shi, Xiaoyu Yu, Guangdong Lan

**Affiliations:** 1 The Higher Educational Key Laboratory for Measuring & Control Technology and Instrumentation of Heilongjiang Province, School of Measurement-Control Technology and Communications Engineering, Harbin University of Science and Technology, Harbin 150080, China; fengqiaohua80@126.com (Q.F.); zhanghuanhuan357@126.com (H.Z.); shiyunbo@hrbust.edu.cn (Y.S.); 2 School of Atmospheric Sciences, Sun Yat-sen University, Guangzhou 519000, China; eeslgd@mail.sysu.edu.cn

**Keywords:** polyaniline/tin dioxide (PANI/SnO_2_), hybrid material, gas sensitivity, ammonia, benzene vapor

## Abstract

A sensor operating at room temperature has low power consumption and is beneficial for the detection of environmental pollutants such as ammonia and benzene vapor. In this study, polyaniline (PANI) is made from aniline under acidic conditions by chemical oxidative polymerization and doped with tin dioxide (SnO_2_) at a specific percentage. The PANI/SnO_2_ hybrid material obtained is then ground at room temperature. The results of scanning electron microscopy show that the prepared powder comprises nanoscale particles and has good dispersibility, which is conducive to gas adsorption. The thermal decomposition temperature of the powder and its stability are measured using a differential thermo gravimetric analyzer. At 20 °C, the ammonia gas and benzene vapor gas sensing of the PANI/SnO_2_ hybrid material was tested at concentrations of between 1 and 7 ppm of ammonia and between 0.4 and 90 ppm of benzene vapor. The tests show that the response sensitivities to ammonia and benzene vapor are essentially linear. The sensing mechanisms of the PANI/SnO_2_ hybrid material to ammonia and benzene vapors were analyzed. The results demonstrate that doped SnO_2_ significantly affects the sensitivity, response time, and recovery time of the PANI material.

## 1. Introduction

Gas-sensitive materials are a class of functional materials whose resistance changes with the surrounding gas environment. Currently, sensitive materials used as gas sensors can be divided into two categories: inorganic materials and organic materials. For inorganic sensing materials such as tin dioxide (SnO_2_), ZnO, Fe_2_O_3_, and TiO_2_, the preparation method is simple, the cost of raw materials is low, and some have been commercialized. Nevertheless, the main limitations of such sensors are their high operating temperatures (200–450 °C) and poor selectivity [1,2]. Organic sensitive materials such as polypyrrole, polyaniline (PANI), and metal phthalocyanine have the advantages of lower operating temperatures and simple preparation methods; however, they have the limitations of poor processing, long response and recovery times, and poor selectivity [3,4].

To compensate for a single organic or inorganic sensitive material defect, and to fully facilitate the functional advantages of organic/inorganic hybrid materials, many scholars have studied the preparation methods and sensitive characteristics of organic/inorganic hybrid materials. Lin et al. [5] prepared an organic/inorganic hybrid film for the first time in 1991 using sol-gel and spin-coating methods, and studied its gas sensitivity. The results showed that the hybrid membranes had low cross-selectivity, low dependence on temperature and humidity, and good reversibility. Since the beginning of this century, the gas sensing of some polyaniline hybrid materials has been reported. These hybrid materials include NO_2_ and triethylamine with SnO_2_-ZnO/PANI [6,7], H_2_S, CO, NH_3_, and volatile organic compounds with PANI/SnO_2_ [8,9,10,11,12,13,14,15], *E. coli* bacteria, acetone, ammonia, ethanol, and CO with PANI/TiO_2_ [16,17,18,19,20,21,22,23,24], and NH_3_ with PANI/MoO_3_ and PANI/nano-In_2_O_3_ [25,26,27,28]. Recently, numerous studies have been reported on the benzene gas sensing behavior of these hybrids such as polyaniline/graphene [29,30]; however, reports on the detection of benzene gases at low concentrations are limited. In this study, a PANI/SnO_2_ hybrid material was prepared using a chemical oxidative polymerization method and characterized by scanning electron microscopy (SEM) and differential thermogravimetric analysis (DTA). The gas sensitivities to ammonia and benzene vapors were tested at 20 °C.

## 2. Materials and Methods

**Figure 1:**
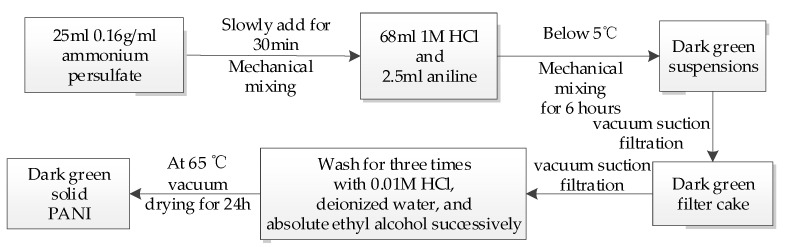
Preparation process flow chart of polyaniline (PANI).

In the experiment, double-distilled 2.5 mL of aniline was dissolved in 68 mL of 1 M hydrochloric acid (HCl) under mechanical agitation. Ammonium persulfate (25 mL) was slowly added dropwise at 0.16 g/mL (a molar ratio of aniline to ammonium persulfate of 1:1) for 30 min. The reaction was allowed to proceed for 6 h at temperatures below 5 °C. When the leach through vacuum suction filtration after the reaction stopped, the filter cake was washed three times using 0.01 M HCl, deionized water, and absolute ethanol, respectively. It was then dried under vacuum at 65 °C for 24 h to obtain PANI. The preparation process is illustrated in Figure 1.

**Figure 2:**
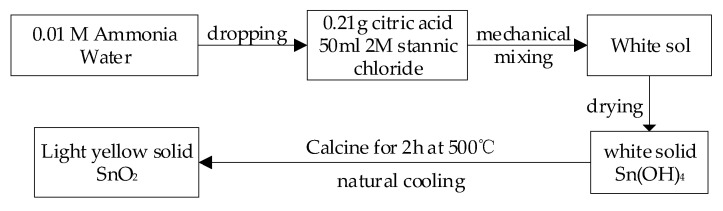
Preparation process of SnO_2_.

We dissolved 3.5 g of tin tetrachloride (stannic chloride) in 50 mL of deionized water and added 0.21 g of citric acid as a dispersant. After the tin tetrachloride and citric acid were completely dissolved, the solution was titrated with ammonia (0.01 M) until the pH of the solution was 12. The sol obtained was dried and calcined for 2 h at 500 °C. It was allowed to cool naturally, and SnO_2_ was obtained. Figure 2 shows the preparation process of SnO_2_.

The PANI and SnO_2_ were mixed in proper proportions and ground for more than 30 min to prepare the PANI/SnO_2_ hybrid material.

**Figure 3:**
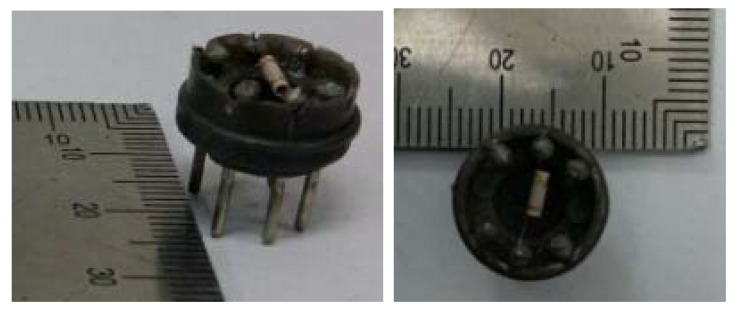
Photograph of a fabricated sensor.

In the experiments, an indirectly heated gas sensor was fabricated using a conventional production process. The PANI and PANI/SnO_2_ gas-sensitive materials and the 1-methyl-2-pyrrolidinone solvent were fully ground and coated on the surface of a ceramic tube with electrodes. We placed it in the PANI gas sensor and PANI/SnO_2_ gas sensor at 20 °C. The gas sensing characteristics of the prepared gas sensors were tested using a static test system. Photographs of the fabricated sensor are shown in Figure 3, where the dimensions of the sensor are ~13 mm × 14 mm, and the thickness of the coating is approximately 0.1 μm.

## 3. Results and Discussion

### 3.1. Characterization of Materials

**Figure 4:**
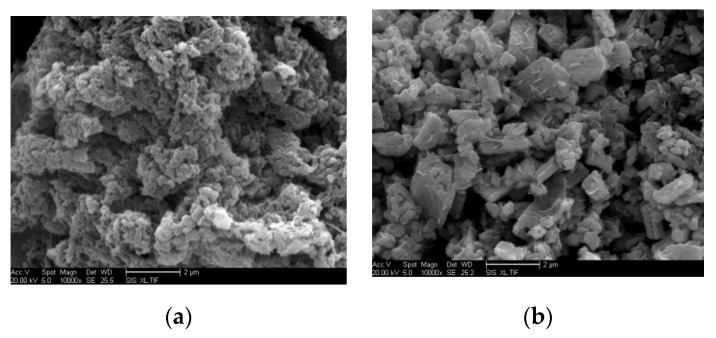
Scanning electron microscopy (SEM) photographs of prepared materials. (**a**) PANI; (**b**) PANI/SnO_2_.

The scanning electron microscope (SEM) photographs of the prepared materials are shown in Figure 4. Figure 4a shows a 10,000 times magnification SEM photograph of PANI, which was indirectly heated by the gas sensor. The PANI particles were dense and had a macromolecular chain structure. Figure 4b shows a 10,000 times magnification SEM photograph of PANI/SnO_2_. As shown in the latter figure, the block or sheet particles are SnO_2_, while the smaller particles are PANI. The nanosheet SnO_2_ was embedded in the polyaniline; the SnO_2_ particles and PANI particles were uniformly mixed together. After the doping of SnO_2_, the PANI particles became smaller; however, the gaps between the particles remained, which helps with the adsorption of gas molecules.

**Figure 5:**
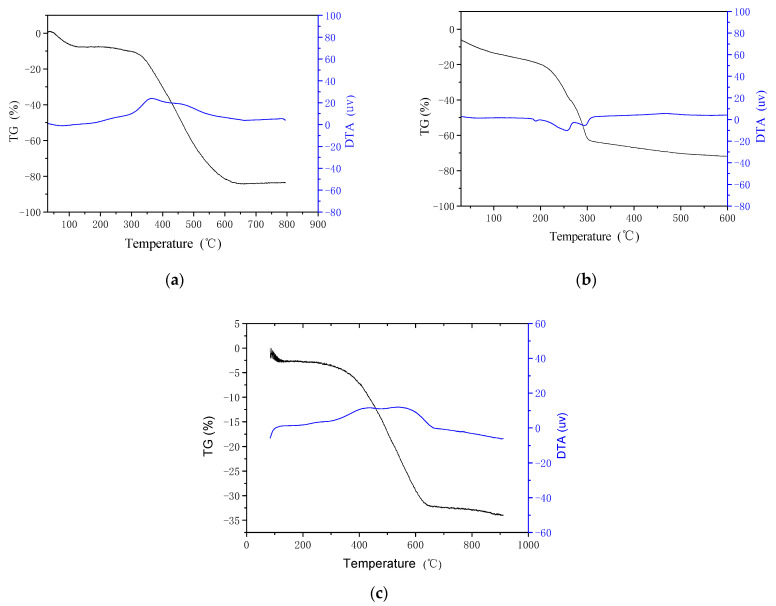
Thermogravimetric Analysis-Differential Thermal Analysis **(**TG-DTA) curves. (**a**) PANI; (**b**) tin hydroxide; (**c**) PANI/SnO_2_.

It is generally known that most organic matter, like PANI, have poor thermal stability. Therefore, to measure the thermal decomposition temperature of PANI, we conducted a differential thermal gravimetric analysis experiment on PANI using a ZCT-B-type differential thermal gravimetric analyzer. The measured TG-DTA (Thermogravimetric Analysis-Differential Thermal Analysis) curves shown in Figure 5 were obtained at a scanning speed of 10 °C/min and show the thermogravimetry (TG) and DTA as a function of temperature. In the TG-DTA curves, the black curve is that of the TG, and the blue curve is that of the DTA.

According to the TG-DTA curve of PANI (as shown in Figure 5a), PANI experienced continuous weight loss from the time that heating started. Moreover, it has two evident asymmetric endothermic peaks: the first is between 250 and 420 °C, and the second is not significant. Therefore, the thermal decomposition of PANI can be divided into three stages: (1) The first weight loss occurs below 100 °C and is caused by water molecules breaking away from the PANI; (2) the weight loss between 100 °C and ~250 °C is due to the fact that the HCl dopant is removed from the PANI due to the low removal temperature of HCl, therefore, there was no decomposition process for the weight loss of the removal agent; and (3) the weight loss between 250 °C and ~420 °C is due to the decomposition of some polymer chains and a small amount of oligomer in the material. The weight loss between 420 °C and 550 °C is due to the degradation of the macromolecular chains. The polymer completely decomposed at temperatures over 590 °C. Both the TG and DTA curves remained constant for temperatures above 590 °C. This indicates that PANI begins to decompose at 250 °C.

To obtain the optimum calcination temperature for tin hydroxide, we performed an experiment using tin hydroxide differential thermal gravimetric analysis. The measured TG-DTA curves for tin hydroxide are shown in Figure 5b. The figure shows that the thermal decomposition of tin hydroxide can be divided into two stages: (1) The first weight loss below 200 °C was caused by the evaporation of deionized water, citric acid, and aqueous ammonia, and by complete decomposition of unreacted sodium hydroxide; and (2) the weight loss between 300 °C and ~800 °C was due to the breakaway of the hydroxyl ion in tin hydroxide. Eventually, SnO_2_ is produced. The calcination temperature of tin hydroxide is 500 °C, according to the tin hydroxide DTA curve.

Figure 5c shows the TG-DTA curve of the PANI/SnO_2_ hybrid material. Its shape was similar to that of PANI; the two endothermic peaks are relatively symmetrical and clear at temperatures between 350 °C and ~450 °C and between 500 °C and ~650 °C, respectively. The hybrid material showed almost no weight loss below 250 °C. When compared to Figure 5a, it is clear that the thermal stability of PANI improved in the hybrid material and the hybrid is more suitable as a gas-sensitive material.

**Figure 6:**
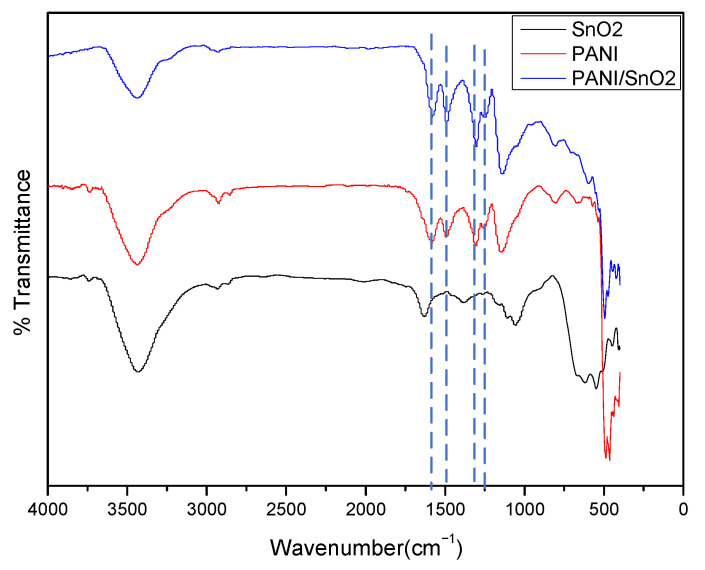
Fourier transform infrared (FTIR) spectra of PANI, SnO_2_, and PANI/SnO_2_.

The structural aspects of the PANI, SnO_2_, and PANI/SnO_2_ hybrid materials were analyzed by Fourier transform infrared (FTIR) spectroscopy, as shown in Figure 6. The FTIR spectra of PANI (polyaniline doped with HCl) shows the important characteristic peaks at 1583 cm^−1^ (C=C quinoid rings), 1487 cm^−1^ (C=C, benzenoid rings), 1302 cm^−1^ (C–H in-plane bending), and 1252 cm^−1^ (C–N in-plane bending) [29]. The major characteristic peaks of PANI (in red) were slightly shifted in the hybrid spectra (in blue), which suggests that the addition of SnO_2_ does not change the intrinsic structure of PANI.

### 3.2. Test of Gas Sensing Properties

In the experiments, we tested the gas sensing properties of the PANI and PANI/SnO_2_ gas sensors to ammonia and benzene vapors.

**Figure 7:**
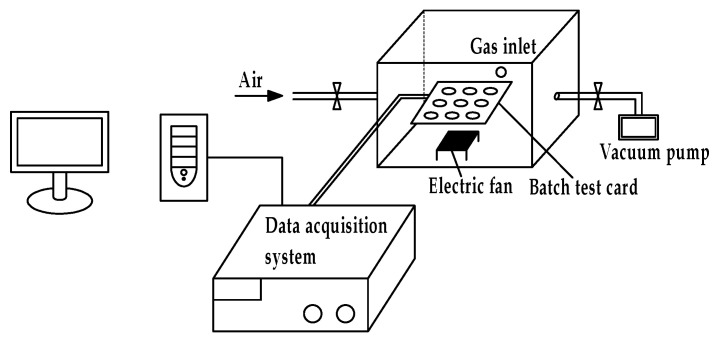
The test device diagram of the gas sensor.

The sensor performance test system consisted of four components: a gas test box, a data acquisition system (Agilent 34901A), a computer, and an exhaust device. During the test, the sensors were inserted into a batch test card in the gas test box. The test gas was introduced through the gas inlet on the gas test box and evenly mixed with the air using an electric fan. When the gas-sensitive film on the sensor came into contact with the test gas, the resistance of the sensor changed. The data acquisition system collected the resistance values of the sensors at a frequency of 20 ms/time, and the computer displayed and saved the data in real time. After completion of the test, the valves were opened on both sides of the test box and a vacuum pump used to quickly remove the test gas. A diagram of the test device is shown in Figure 7.

#### 3.2.1. Ammonia Gas Sensing Properties

**Figure 8:**
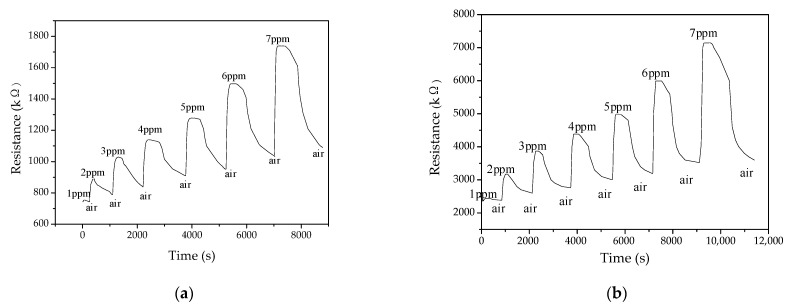
Gas sensor response curves for different ammonia concentrations. (**a**) PANI; (**b**) PANI/SnO_2_.

Figure 8 shows the gas sensing response curves of the PANI and PANI/SnO_2_ gas sensors to ammonia at 20 °C and a relative humidity of 40 ± 5%. Figure 8a,b shows the response curves of the PANI and PANI/SnO_2_ gas sensors, respectively. The resistances of both gas sensors increased with increasing ammonia concentration. In addition, the gas sensor resistances did not return completely to the initial resistance values because the ammonia was not completely desorbed.

**Figure 9:**
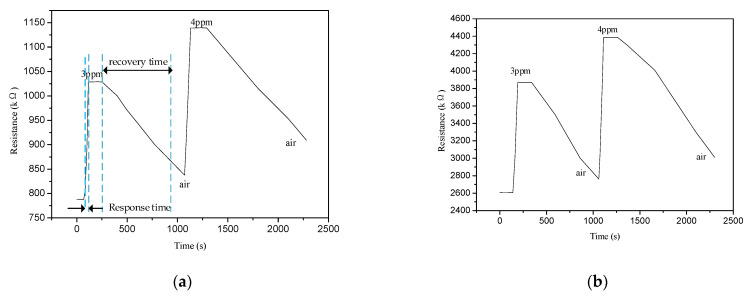
Gas sensor ammonia response and recovery curves. (**a**) PANI; (**b**) PANI/SnO_2_.

The response and recovery characteristic curves of both devices for 3 and 4 ppm ammonia are shown in Figure 9. The response time is defined as the time from the sensor first making contact with the ammonia gas to the time at which the sensor resistance reached 90% of the saturated value of resistance in ammonia gas. The recovery time is the time from when the ammonia starts to be rapidly exhausted to the time at which the sensor resistance is restored to 110% of the saturated value of the resistance in clean air.

When the gas sensor makes contact with the gas, the resistance of the gas sensor changes, and the time elapsed until it reaches 90% of the stable value is the response time of the gas sensor. When emptying the gas from the test box, the resistance of the gas sensor gradually returns to the initial resistance in the air, and the time required to return to 90% of the initial resistance value is called the recovery time of the sensor. As shown in Figure 9a, the response time of the PANI gas sensor was ~40 s, and the recovery time was ~960 s. As shown in Figure 9b, the response time of the PANI/SnO_2_ gas sensor at ~44 s was slightly longer than that of the PANI gas sensor. However, the recovery time at ~915 s was shorter. Under the same test conditions, SnO_2_ did not respond to ammonia at 20 °C.

**Figure 10:**
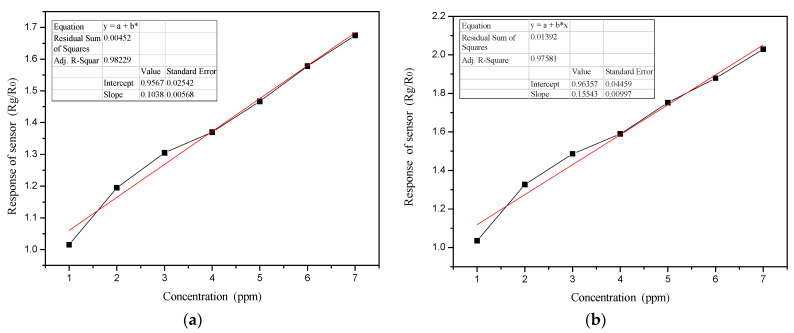
Gas sensor response curves with ammonia concentration. (**a**) PANI; (**b**) PANI/SnO_2_. The asterisk (*) represents a multiplier (x).

Figure 10 shows the change in the PANI and PANI/SnO_2_ gas sensor response curves with ammonia concentration. The response of the sensor, S, is defined as the ratio of the gas sensor resistance in detecting the gas, R_g_, to the resistance in clean air, R_0_; that is, S = R_g_/R_0_.

As shown in Figure 10, the responses of the PANI and PANI/SnO_2_ gas sensors increased with increased ammonia concentration; moreover, they showed a good linear relationship. The linear trend line equation of the PANI sensor was y = 0.9567 + 0.1038x, the correlation coefficient was 0.9911, while the linear trend line equation of the PANI/SnO_2_ sensor was y = 0.96357 + 0.15543x, and the correlation coefficient was 0.9878.

#### 3.2.2. Ammonia Sensing Mechanism

The above experimental results at 20 °C indicate that there is almost no difference in the ammonia sensing properties of the PANI and PANI/SnO_2_ gas sensors. The addition of SnO_2_ to the hybrid material reduced the recovery time slightly, which indicated that the response to ammonia gas of the PANI and PANI/SnO_2_ gas sensors depends more on PANI. The conductivity of PANI is mainly due to proton acid doping, which is improved in this study by HCl doping. Ammonia is alkaline. Furthermore, ammonia molecules are smaller than PANI molecules and have a stronger affinity for acids. When sensitive materials are in contact with ammonia, it is easy to capture the doping acid combined with polyaniline amine. Then, the conductivity of PANI decreases because of the loss of part of the proton acid doping, and the resistance of the sensor increases. In contrast, when pure air is piped in to carry out desorption, the ammonia adsorbed by the PANI molecules is desorbed. The ammonia released the doping acid, and PANI was again obtained by doping with the released acid. Accordingly, the conductivity increased and the resistance decreased to close to the initial value.

#### 3.2.3. Benzene Vapor Gas Sensing Properties

**Figure 11:**
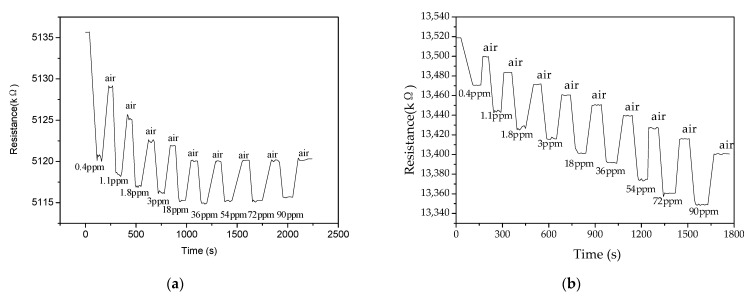
Gas sensor response curves at different benzene vapor concentrations. (**a**) PANI; (**b**) PANI/SnO_2_.

Figure 11a,b shows the response and recovery curves at 20 °C and a relative humidity of 40 ± 5% of the PANI and PANI/SnO_2_ gas sensors, respectively, for different concentrations of benzene vapor.

Figure 11 shows that the resistances of the PANI and PANI/SnO_2_ gas sensors decreased with increased benzene vapor concentrations. As shown in Figure 11a, the resistance value of the PANI gas sensor no longer decreased for concentrations of benzene vapor greater than 36 ppm. This indicates that the PANI gas sensor cannot detect benzene vapor at concentrations greater than 36 ppm. As Figure 11b shows, the resistance of the PANI/SnO_2_ gas sensor also decreased with increased concentrations of benzene vapor up to 90 ppm. It therefore has a larger range of detection than the PANI gas sensor. In the literature [29], the range of benzene gas concentration has been reported as being between 0 and 22,000 ppm, and was used mainly for the detection of high concentrations of benzene gas. This study, however, has detected much lower concentrations of benzene vapor. The range of benzene gas concentration of PANI/SnO_2_ is compared with the literature [29] in Table 1.

**Figure 12:**
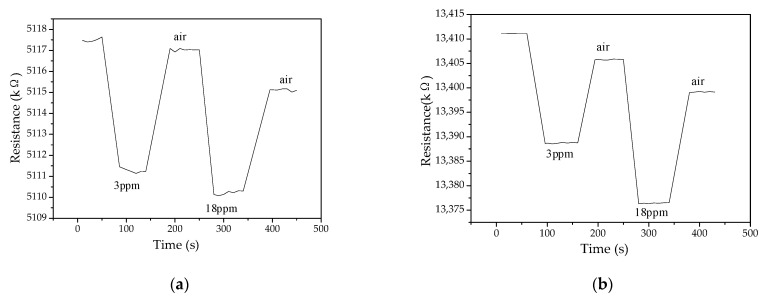
Gas sensor benzene vapor response and recovery curves. (**a**) PANI; (**b**) PANI/SnO_2_.

The PANI and PANI/SnO_2_ gas sensor benzene vapor response and recovery curves are shown in Figure 12a,b, respectively, for concentrations of 3 ppm and 18 ppm.

Figure 12 shows that the response and recovery times of the PANI and PANI/SnO_2_ gas sensors are short. The response time of the PANI gas sensor was 33 s, while the recovery time was ~73 s (see Figure 12a). The response time of the PANI/SnO_2_ gas sensor was ~33 s, while the recovery time was also ~33 s. Compared to the PANI gas sensor, the PANI/SnO_2_ gas sensor had the same response time, but a shorter recovery time (see Figure 12b). However, under the same test conditions, the SnO_2_ gas sensor did not respond to benzene vapor at 20 °C. Thus, the addition of SnO_2_ to PANI reduced the recovery time of the sensor.

**Figure 13:**
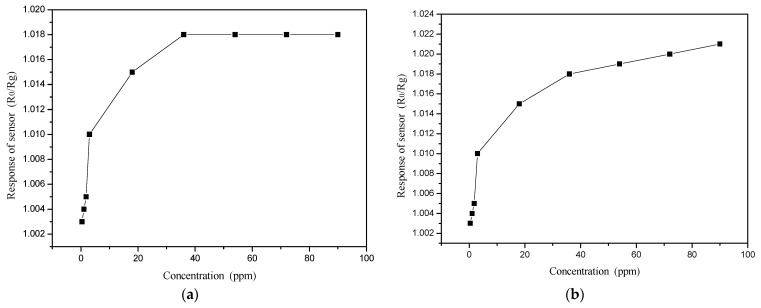
Gas sensor response curves with benzene vapor concentration. (**a**) PANI; (**b**) PANI/SnO_2_.

The change in the PANI and PANI/SnO_2_ gas sensor response curves with benzene vapor concentration are shown in Figure 13a,b, respectively.

As shown in Figure 13, the response of the PANI and PANI/SnO_2_ gas sensors increased with increased benzene vapor concentration. As shown in Figure 13a, the response of the PANI gas sensor was constant for benzene vapor concentrations greater than 36 ppm. Figure 13b shows that the response of the PANI/SnO_2_ gas sensor increased continuously with increased benzene vapor concentration, showing a linear relationship at concentrations >36 ppm. Thus, the PANI/SnO_2_ hybrid material was suitable to detect a wide range of benzene vapor concentrations.

#### 3.2.4. Benzene Vapor Sensing Mechanism

At 20 °C, when the PANI/SnO_2_ hybrid material adsorbs benzene vapor, the resistance of the sensor decreases with increased benzene vapor concentration. Moreover, at 20 °C, the single SnO_2_ gas sensor is insensitive to benzene vapor, which indicates that PANI plays a significant role in the hybrid material. The concentration limit of measurement of the PANI/SnO_2_ hybrid material for benzene vapor is higher than that of PANI. In addition, the recovery time of the PANI/SnO_2_ hybrid material is half that of PANI, which indicates that SnO_2_ also plays an important role in the process of gas detection.

The response of PANI to benzene vapor can be explained by hydrogen bonding theory. When the PANI/SnO_2_ hybrids come into contact with benzene vapor, the internal hydrogen bonds of the hybrids break and the stress decreases. Accordingly, more conductive strands form, and the resistance decreases. When the hybrids are desorbed through clean air, the internal bonds regroup, the hybrid material structure returns to its initial state, and the resistance increases to its initial value.

Furthermore, because PANI is a p-type semiconductor and SnO_2_ is an n-type semiconductor, a combination of these forms a heterojunction. Migration between particles in the heterojunction causes a positively charged depletion layer to form on the SnO_2_ surface, which can reduce the activation energy and enthalpy of the physically adsorbed gas. Therefore, the range of benzene vapor detection is expanded, and the recovery time is reduced.

## 4. Conclusions

In our experiments, we tested the gas sensing characteristics of both a PANI material and a PANI/SnO_2_ hybrid material for 1–7 ppm of ammonia and 0.4–90 ppm of benzene vapor at 20 °C and a relative humidity of 40 ± 5%.

The results showed that the response time of the PANI/SnO_2_ materials was slightly longer than that of the PANI material; however, the recovery time was shorter. The response time was ~44 s, and the recovery time was ~915 s. The response of the PANI/SnO_2_ hybrid material showed a linear relationship with an ammonia concentration of 1–7 ppm. The linear trend line equation of the PANI sensor was y = 0.9567 + 0.1038x, the correlation coefficient was 0.9911; the linear trend line equation of the PANI/SnO_2_ sensor was y = 0.96357 + 0.15543x, and the correlation coefficient was 0.9878. We conclude that SnO_2_ has little effect on the ammonia gas sensing properties of PANI.

For benzene vapor, the results showed that the concentration limit of detection of the single PANI material for benzene vapor was 36 ppm, whereas that of the PANI/SnO_2_ hybrid material was beyond the range tested. The recovery times of the PANI/SnO_2_ hybrid material were shorter. The response time was ~33 s, and the recovery time was ~33 s. We conclude that the PANI/SnO_2_ hybrid material for benzene vapor has shorter recovery times and a wider range than PANI.

## Figures and Tables

**Table 1:** Range of benzene gas concentration.

Test Gas	PANI	PANI/SnO_2_	[29]
Benzene gas concentration	0–36 ppm	0–90 ppm	0–22,000 ppm

## Data Availability

Not applicable.
